# Health sector advocacy for repurposing agricultural investments affecting fruits, vegetables and legumes

**DOI:** 10.2471/BLT.24.292201

**Published:** 2025-04-08

**Authors:** Erica Reeve, Daniel Mason-D'Croz, Anne Marie Thompson Thow

**Affiliations:** a Global Centre for Preventive Health and Nutrition, Institute for Health Transformation, Deakin University, 1 Gheringhap St, Geelong VIC 3220, Australia.; b Department of Global Development, Cornell University, Ithaca, United States of America.; c Menzies Centre for Health Policy, University of Sydney, Sydney, Australia.

## Abstract

Increasing population intake of fruits, vegetables and legumes could reduce diet-related mortality. The World Health Organization recommends that countries adopt fiscal tools to address the relative affordability of healthy foods, including through taxes and subsidies. Most global agricultural subsidy support has been tied to the production of specific commodities, predominately grains. Heavily embedded financial and regulatory focus on a narrow range of commodities has encouraged monocropping and intensive farming at the expense of dietary diversity. To address this issue, the United Nations recommends that countries phase out distortive policies and subsidies, and repurpose these with more efficient and equitable measures. This would provide an opportunity for the health policy community to engage on the investment needed to promote production, supply and demand for fruit, vegetables and legumes. This article supports this engagement, by describing the current policy context for agricultural subsidies and some of the specific policy options for increasing fruit, vegetable and legume production, supply and demand. The article outlines ways through which the health policy community can support the development of a repurposing policy agenda for fruit, vegetables and legumes by building awareness of the benefits of such an agenda; strategically engaging across sectors to develop a cohesive package of policy measures for increasing fruit, vegetable and legume production, supply and demand; and engaging with agriculture and other sectors to navigate the complexities of a repurposing agenda, ensuring a range of sectoral concerns are addressed.

## Résumé

L’augmentation de la consommation de fruits, de légumes et de légumineuses au sein de la population pourrait réduire la mortalité liée à l’alimentation. L’Organisation mondiale de la santé recommande aux pays d’adopter des outils fiscaux pour remédier à l’accessibilité relative des aliments sains, notamment par le biais de taxes et de subventions. La plupart des subventions agricoles mondiales ont été liées à la production de denrées spécifiques, principalement des céréales. Cette orientation financière et réglementaire fortement ancrée sur un éventail limité de produits de base a encouragé la monoculture et l’agriculture intensive au détriment de la diversité alimentaire. Pour résoudre ce problème, les Nations unies recommandent aux pays d’éliminer progressivement les politiques et les subventions perturbatrices et de les remplacer par des mesures plus efficaces et plus équitables. Ce faisant, la communauté liée aux politiques de la santé aurait l’occasion d’encourager les investissements nécessaires pour promouvoir la production, l’offre et la demande de fruits, de légumes et de légumineuses. Le présent article soutient cet encouragement en décrivant le contexte politique actuel des subventions agricoles et certaines des options politiques spécifiques pour augmenter la production, l’offre et la demande de fruits, de légumes et de légumineuses. Il décrit les moyens permettant à la communauté liée aux politiques de la santé de soutenir le développement d’un programme politique de réorientation en matière de fruits, de légumes et de légumineuses: sensibilisation aux avantages d’un tel programme; engagement stratégique intersectoriel pour élaborer un ensemble cohérent de mesures politiques visant à augmenter la production, l’offre et la demande de fruits, de légumes et de légumineuses; et accord avec l’agriculture et d’autres secteurs pour explorer les complexités d’un programme de réorientation, en veillant à prendre en compte une série de préoccupations sectorielles.

## Resumen

El aumento del consumo de frutas, hortalizas y legumbres por parte de la población podría reducir la mortalidad asociada a la alimentación. La Organización Mundial de la Salud recomienda que los países adopten herramientas fiscales para mejorar la asequibilidad relativa de los alimentos saludables, lo que incluye el uso de impuestos y subvenciones. La mayor parte del apoyo mundial en forma de subvenciones agrícolas se ha vinculado a la producción de productos específicos, principalmente cereales. La orientación financiera y regulatoria, fuertemente arraigada y centrada en un grupo limitado de productos básicos, ha fomentado el monocultivo y la agricultura intensiva, en detrimento de la diversidad alimentaria. Para abordar esta cuestión, las Naciones Unidas recomiendan que los países eliminen progresivamente las políticas y subvenciones distorsionadoras y las reorienten hacia medidas más eficientes y equitativas. Esta propuesta constituiría una oportunidad para que el sector sanitario participara en las decisiones de inversión necesarias para promover la producción, el suministro y la demanda de frutas, hortalizas y legumbres. Este artículo respalda esta participación, al describir el contexto político actual en materia de subvenciones agrícolas y algunas de las opciones específicas de política para aumentar la producción, el suministro y la demanda de frutas, hortalizas y legumbres. Asimismo, expone diversas vías mediante las que el sector sanitario puede contribuir al desarrollo de una agenda política de reorientación, sensibilizando sobre sus beneficios; promoviendo una colaboración estratégica intersectorial para desarrollar un paquete coherente de medidas destinadas a aumentar la producción, el suministro y la demanda de estos alimentos; y participando con el sector agrícola y otros sectores para afrontar las complejidades de una agenda de reorientación que tenga en cuenta las distintas preocupaciones sectoriales.

## ملخص

إن الزيادة في استهلاك السكان من الفواكه والخضراوات والبقوليات، يمكن أن تُقلل من الوفيات المرتبطة بالنظام الغذائي. تُوصي منظمة الصحة العالمية الدول باعتماد أدوات مالية لمعالجة القدرة النسبية على تحمل تكاليف الأغذية الصحية، بما في ذلك من خلال الضرائب والإعانات. وقد ارتبط معظم دعم الدعم الزراعي العالمي بإنتاج سلع محددة، وبخاصة الحبوب. إن التركيز المالي والتنظيمي المتأصل بعمق على مجموعة محدودة من السلع، قد أدى لتشجيع زراعة المحصول الواحد، والزراعة المكثفة على حساب التنوع الغذائي. ولمعالجة هذه المشكلة، تُوصي الأمم المتحدة الدول بأن تقوم بالتخلص التدريجي من السياسات والإعانات المعيبة، وإعادة توجيهها مع إجراءات أكثر فعالية ومساواة. إن ذلك من شأنه أن يتيح الفرصة لمجتمع السياسات الصحية، للمشاركة في الاستثمار اللازم لتعزيز الإنتاج، والعرض والطلب، على الفواكه والخضراوات والبقوليات. تدعم هذه المقالة هذه المشاركة، عن طريق وصف السياق السياسي الحالي للإعانات الزراعية، وبعض من خيارات السياسات المحددة لزيادة إنتاج الفواكه والخضراوات والبقوليات، والعرض والطلب عليها. توضح المقالة الطرق التي يمكن من خلالها لمجتمع السياسة الصحية دعم تطوير جدول عمل لسياسة إعادة توجيه الفواكه والخضروات والبقوليات، عن طريق بناء الوعي بفوائد جدول الأعمال هذا؛ والمشاركة الاستراتيجية عبر القطاعات لتطوير حزمة متماسكة من الإجراءات السياسية لزيادة إنتاج الفواكه والخضروات والبقوليات، والعرض والطلب عليها؛ والمشاركة مع القطاع الزراعي والقطاعات الأخرى للتغلب على تعقيدات جدول أعمال إعادة التوجيه، وضمان التعامل مع مجموعة من المخاوف بالقطاع.

## 摘要

增加人们的水果、蔬菜和豆类摄入量可降低饮食相关死亡率。世界卫生组织建议各国利用财政工具（包括通过征收税款和提供补贴）来解决健康食物的相对可负担性问题。大多数全球农业补贴支持都与特定商品（主要是谷物）的生产挂钩。深度整合的金融政策和高度渗透的监管条例重点支持范围较小的一系列商品，并鼓励推行损害饮食多样性的单一作物种植和集约农业。 为解决这一问题，联合国建议各国逐步取消扭曲市场的政策和补贴方案，并采取更加有效和公平的措施来进行调整。此举确保卫生政策共同体有机会参与必要投资， 从而提高水果、蔬菜和豆类的产量并刺激供需健康发展。本文描述了当前为提高水果、蔬菜和豆类的产量并刺激供需健康发展而推行的农业补贴措施和一些具体政策方案的政策背景，以支持卫生政策共同体参与投资。本文概述了卫生政策共同体可采取以下方法来支持制定水果、蔬菜和豆类相关政策调整议程：宣传此类议程的好处；战略性地跨部门参与制定一系列具有连贯性的政策措施，以提高水果、蔬菜和豆类的产量并刺激供需健康发展；以及与农业和其他部门合作，以应对政策调整议程方面的复杂问题，从而确保解决各部门的相关问题。

## Резюме

Увеличение потребления населением овощей, фруктов и бобовых способно снизить смертность, связанную с неправильным питанием. Всемирная организация здравоохранения рекомендует странам использовать фискальные методы для обеспечения относительной доступности здорового питания, в том числе действовать через налоги и субсидии. На глобальном уровне большинство сельскохозяйственных субсидий направлены на выращивание определенных культур, преимущественно зерновых. Из-за того, что финансирование и законодательство в значительной мере сосредоточены на узком ассортименте производимых продуктов, сложилась практика поощрения монокультур и интенсивного фермерства в противовес разнообразию пищевой продукции. Чтобы изменить сложившуюся ситуацию, Организация Объединенных Наций рекомендует странам постепенно отказаться от применения политики и субсидий, которые оказывают негативное воздействие, и перенаправить их в более эффективное русло с равномерным распределением ресурсов. Тем самым группы, отвечающие за политику в области здравоохранения, получат возможность участвовать в инвестициях, необходимых для поощрения выращивания, поставки и создания спроса на фрукты, овощи и бобовые. Данная статья выступает в поддержку такого подхода, описывая текущий контекст политик в области сельскохозяйственных субсидий и некоторые конкретные политические меры для увеличения производства, поставок и спроса на фрукты, овощи и бобовые культуры. В статье кратко изложены способы, которыми группы, отвечающие за политику в области здравоохранения, могут поддержать разработку и выдвижение на повестку дня политических мер, касающихся потребления фруктов, овощей и бобовых, расширяя осведомленность о пользе таких мер и содействуя скоординированным действиям различных секторов с целью разработки единого пакета действий. Эти усилия могут включать сотрудничество с сельскохозяйственным сектором и другими отраслями экономики в преодолении сложностей, связанных с перераспределением инвестиций и решением целого ряда проблем в этих отраслях.

## Introduction

The 2021 United Nations (UN) Food Systems Summit^1^ brought increased attention to the hidden costs of agrifood systems on health, environments and livelihoods. Growing evidence exists of the need to address major inequities in food systems that have become key drivers of global malnutrition.^2^ Today, over 3.1 billion people around the world are unable to afford a healthy diet,^2^ and consumption of whole grains, nuts and seeds, vegetables and fruits has decreased relative to the intake of processed foods high in sodium, sugar and fat.^3^ These changes have contributed to 149 million children around the world experiencing stunting while 45 million experience wasting, and a further 39 million are overweight.^4^ At the same time, 2.5 billion adults around the world are classified as overweight or obese.^5^ Fruits and vegetables are important sources of micronutrients, and their increased consumption is associated with reductions in the prevalence of many noncommunicable diseases. In 2017, inadequate consumption globally contributed to 3.9 million premature deaths.^6^ Consumption of legumes is inversely associated with risk of heart disease and diabetes.^7^ As such, the World Health Organization (WHO) recommends that countries adopt policy measures to increase population intake of fruits, vegetables and legumes.^8^^,^^9^ However, governments have tended to overemphasize the role of education or to offer incentives for behaviour change as a means for promoting these foods^10^^–^^12^ instead of policy approaches that directly shape demand by addressing their availability and affordability.^13^

WHO recommendations to adopt fiscal tools to address the relative affordability of healthy diets (including taxes and subsidies) have led to a rise in the use of taxes to disincentivize unhealthy food consumption over the past 20 years.^14^ In contrast, uptake of subsidies that aim to reduce the cost of healthy foods, which remain more expensive than unhealthy foods, has been limited.^2^^,^^14^ This disparity has often been due to entrenched political support for existing subsidies (which tend not to be nutritionally oriented) as well as budgetary limitations. The public health community could provide relevant insights and advice; however, they often lack power or opportunity to advocate in the interests of nutrition or equity when decisions affecting food production, manufacture and trade are made. Additionally, health policy-makers often lack familiarity with the policies outside their mandate that shape agriculture and food, and thus face challenges providing policy advice on exactly what needs to be subsidized for nutrition, and how.^15^^–^^17^

In this article, we outline the current policy context for agricultural subsidies, and how it is evolving.^18^ Our aim is to build knowledge among the health policy community to engage with agriculture on the ways that fiscal and market incentives (that is, subsidies) could better service both agricultural and health policy objectives, and provide a series of steps policy-makers can take to support this engagement.

## Agricultural subsidy context 

Governments invest in agriculture and food systems through fiscal and market incentives, including grants and subsidies offered directly to producers, processors or retailers, border measures, market controls, tax holidays and general service support mechanisms (Table 1).^2^ These policy measures have played a critical role in social and economic development by incentivizing increased food productivity and exports, and promoting food sufficiency.^20^ However, about 70% of global agricultural subsidies have been tied to the production of specific commodities,^18^ predominately grains (mostly rice, wheat and maize), sugar, dairy, beef and oil crops used in livestock feed,^2^^,^^20^ aligning with the conventional perspective that these are important for food sufficiency and export trade.^2^^,^^18^^,^^20^^,^^21^ These subsidies have contributed to increasing calorie supply; however, incentivizing the production and consumption of a more limited range of foods has introduced a bias that makes these foods relatively cheaper compared to non-subsidized varieties, and has incentivized the development of value chains (such as food processing) based on these cheap inputs. This context has contributed to a food system that supplies and utilizes a narrower range of commodities, contributing to less diverse diets.^2^^,^^20^ Commodity-specific agricultural and food systems subsidies have also contributed to increased monoculture and intensive farming, including overuse of inputs (such as agrochemicals) or natural resources (such as water), causing widespread environmental damage.^2^^,^^18^^,^^22^ Traditionally, producer subsidies have also been regressive, favouring larger farms more capable of managing grant administration and production requirements.^2^^,^^19^

**Table 1:** Implications of current food and agricultural subsidy instruments for fruit, vegetables and legumes

Typology of fiscal and market policies	How these policies are operationalized	Negative implications for fruit and vegetables
Price incentives: border measures	Tariffs, tariff-rate quotas, taxes, export or import bans or licensing, export subsidies or credits, licensing fee exemptions	• Tariff rates by nature distort food production and consumption.^18^ High export tariffs on fruits and vegetables may disincentivize production and high import tariffs may reduce affordability • Non-tariff measures are harder for fruit and vegetables to comply with (such as phytosanitary testing) • Trade regulations inhibit public health policy-making space
Price incentives: market controls	Administered food prices, minimum producer price policies, fixed price, price controls, public stockholding and reserves	• Create price incentives for production and consumption for small selection of commodities^18^ • Price ceilings on fruit, vegetables and legumes can penalize producers and disincentivize market participation^18^ • Price floors on fruit, vegetables and legumes can protect producers but reduce affordability^2^ • Stockholding of rice and grains skews consumption towards these
Tax incentives	Tax holidays, exemptions for inputs to farming	• Can promote overuse of specific farming inputs (such as fertilizer)^19^ • Regressive for smallholders operating in informal markets
Fiscal subsidy to producers	Transfers of cash, grants or discounts to reduce production costs or increase farm income	• Commodity-specific subsidies promote monoculture and intensive farming, including overuse of inputs (such as agrochemicals) or natural resources (for example, water)^18^^,^^19^ • Regressive for smallholders and critical for fruit and vegetable production^2^ • Costly to maintain and prone to inefficiency and corruption^19^ • Politically difficult to remove^18^ • Long-term implications for farms and food supply
Fiscal support to consumers: affordability	Safety nets, cash or food transfers, school feeding programmes	• Can drive unsustainable demand of specific commodities and distort consumption patterns
Fiscal support to consumers, processors or traders: price	Subsidies, taxes, price controls	• Taxes can drive up the cost of diets overall^17^ and may not be passed through to consumers^2^ • Consumer subsidies are costly to maintain^20^
Fiscal support: general services	Goods and services that promote improved methods, market access, rural transport and infrastructure	• Can be poorly targeted with indirect benefits to fruit and vegetables

Source: Adapted from Food and Agriculture Organization of the United Nations, 2022.^2^

Distortions caused by commodity-specific subsidies are then exacerbated with differential tariff rates that governments establish to reduce barriers to the trade of staple foods (for example, rice and maize), manufacturing ingredients (such as sugar and vegetable oils) and prepackaged foods that make up the bulk of food trade.^23^ Market controls applied to stabilize food pricing and availability emphasize the protection of non-perishable grain supplies (largely maize, rice, wheat and millet), cooking ingredients (oil, sugar and salt)^2^ and processed or staple foods (canned meat and noodles).^24^ Border and market controls that favour these commodities have limited diversification to other commodities including fruit and vegetables.^2^

In fact, producers of fruit and vegetable commodities receive very little subsidy support, and are rarely the beneficiaries of market incentives.^2^ For legumes, agricultural subsidies favouring cereal and soybean production (largely for soybean oils) have reinforced agri-food pathways (also called lock-ins) towards these products at the expense of legume innovation and cropping.^25^ For fruit and vegetables, which are generally highly perishable until they have been processed,^20^^,^^22^ lack of reliable market access and storage accelerates deterioration in quality, leading to substantial losses being incurred by producers, particularly the smallholders engaged in fruit and vegetable production.^22^^,^^26^ This challenge has contributed to major inefficiencies in fruit, vegetable and legume production, with fruit and vegetables in particular remaining relatively more expensive than other commodities. Exporting producers face additional challenges in meeting non-tariff measures such as sanitary and phytosanitary requirements, which are not always harmonized across countries.^22^ Consequently, the global production and consumption of fruit and vegetables is now insufficient for meeting current or future population needs.^2^^,^^12^

Embedded budget and regulatory priority for export production and trade over domestic consumption, population nutrition and equitable resource distribution is difficult to reverse.^27^ Recent analyses suggest that reductions in commodity-specific agricultural and food system subsidies could negatively impact food production, food prices, farm employment and on-farm income, contributing to increased food insecurity and poverty.^28^ Simply reallocating subsidies currently channelled towards staple commodities to the production of specific fruit, vegetable and legume commodities would increase consumption,^29^ but may perpetuate some of the inequities and power dynamics currently observed in food systems.^18^^,^^28^^,^^30^

Scaling up investment in policies that promote production, supply and demand for fruit, vegetables and legumes is clearly needed. However, for many countries, mobilizing new resources is fiscally challenging. Instead, international agencies such as the Food and Agriculture Organization, the International Fund for Agricultural Development, the United Nations Children’s Fund, the World Food Programme and WHO have proposed that a more promising option would be to phase out distortive incentives and to repurpose them to support the production and supply of nutritious foods.^2^ The UN defines repurposing as “the reduction of agricultural producer support measures that are inefficient, unsustainable and/or inequitable, in order to replace them with support measures that are the opposite.”^18^ Repurposing would entail a wholesale change whereby some of the fiscal incentives currently supporting the agricultural sector are redirected towards the provision of agricultural goods and services that are critical in facilitating the production and supply of a wide range of nutritious food grown in sustainable conditions. Repurposing mitigates many of the negative economic impacts of just phasing out harmful subsidies, instead reconfiguring this support to distribute it across the sector more efficiently and equitably, and to achieve multiple food systems outcomes such as nutrition and health, environment and climate, and socioeconomic development.^2^

The repurposing agenda offers a unique opportunity to promote fruit, vegetables and legumes by advocating for the resourcing of policy measures that better address supply-side constraints, and for strengthening food environment policies that promote demand.^9^ However, fiscal and market incentives currently offered to agriculture and food producers are administered through many policy instruments related to trade and investment, revenue, consumer protection, industry development, environmental protections, livelihood and agrarian development.^2^^,^^30^ Most policy-makers in the current siloed systems lack visibility across food system policies, and have limited understanding of which fiscal and market instruments could lift the production and demand of fruit, vegetables and legumes, or of the impact of these instruments on other outcomes.^16^^,^^31^ Additionally, those governing agricultural support confront different political landscapes, constraining their ability to act on the repurposing agenda.^15^^,^^31^

As the agricultural community considers the implication of a repurposing agenda on current food systems, health policy-makers have a unique opportunity to articulate mechanisms for increasing fruit, vegetable and legume production and supply globally, and for promoting consumption. This article identifies three critical steps to support these efforts. First, health policy-makers must effectively communicate the need for the repurposing of agricultural subsidies to support production of fruit, vegetables and legumes. Second, health policy-makers should support identification of tangible, feasible policy measures, considering both health and agricultural policy imperatives. Third, health policy-makers can support political acceptability by working with the agricultural and other sectors to identify ways these polices could be designed to promote other development benefits and mitigate potential trade-offs.^2^^,^^17^

## Demonstrating the benefits

A first step for engaging on the repurposing agenda is drawing attention to the need for repurposing policies that address fruit, vegetable and legume supply and demand.^18^ Building a shared sense of responsibility for an agenda that supports this objective together with other food systems outcomes might facilitate greater cooperation between the health and agricultural sectors, and thus greater support from across government entities.^15^^,^^17^ The health policy community can use country-level estimates to develop a case that is compelling to agricultural policy-makers, whose primary objective is to contribute to national productivity and export, and to promote industry and rural development.^31^ Inadequate fruit, vegetable and legume consumption, which is a primary dietary risk factor for noncommunicable diseases,^8^ has one of the strongest correlations to morbidity compared with other dietary risks.^8^^,^^32^ The disproportionate impact of these diseases on the working-age population in low- and middle-income countries compromises productivity, economic growth and development.^33^ Any compromise to the labour supply resulting from noncommunicable disease risk has specific implications for agrifood sectors, which are labour-intensive and are already facing declines in workforce related to ageing producer demographics, and urbanization.^34^ Lastly, investments in legume production and consumption has benefits for human health due to their role in gut health and nutrient content, but also for the environment, due to the ecosystem services provided by legumes, and their role in meat displacement.^35^

According to global estimates, repurposing agricultural subsidies towards the production of healthier foods (primarily fruit, vegetables and legumes) could increase consumption of fruit and vegetables by 10% in countries of the Organisation for Economic Co-operation and Development and 5% in other countries, with mortality benefits of increased consumption estimated to generate 12 billion United States dollars in workforce and labour supply worldwide.^28^

Agricultural sectors are increasingly concerned about resource losses and environmental resilience.^31^ Legume production is highly lauded as having environmental co-benefits linked to their ability to counter the impact of nitrate leaching from monocropping systems and to promote soil and structural diversity.^25^ Investments in initiatives that support a transition towards plant-based diets are likely to have benefits on environmental outcomes by moderately reducing greenhouse gas emissions^28^ and reducing water and chemical inputs, where repurposing efforts have been strategically designed to support improved methods to achieve these outcomes.^25^^,^^28^ Additionally, fruit and vegetable production systems are dominated by small-scale producers and vendors (including women), and initiatives to promote their production can have substantial benefits on their livelihoods.

## Defining the policy package

The repurposing agenda provides a much-needed opportunity to counter the impacts of chronic underinvestment in fruit and vegetables together with initiatives to increase demand for their consumption.^2^^,^^22^^,^^36^ Strategic engagement with agricultural policy stakeholders in setting the agenda can ensure repurposing strategies are designed in ways that address multiple development priorities,^17^ for instance by ensuring fruit and vegetables are accessible and affordable to all, and that they are produced and supplied in ways that promote resilience and livelihood outcomes, using methods that are within planetary boundaries.^18^

### Production and supply

**Fig. 1:**
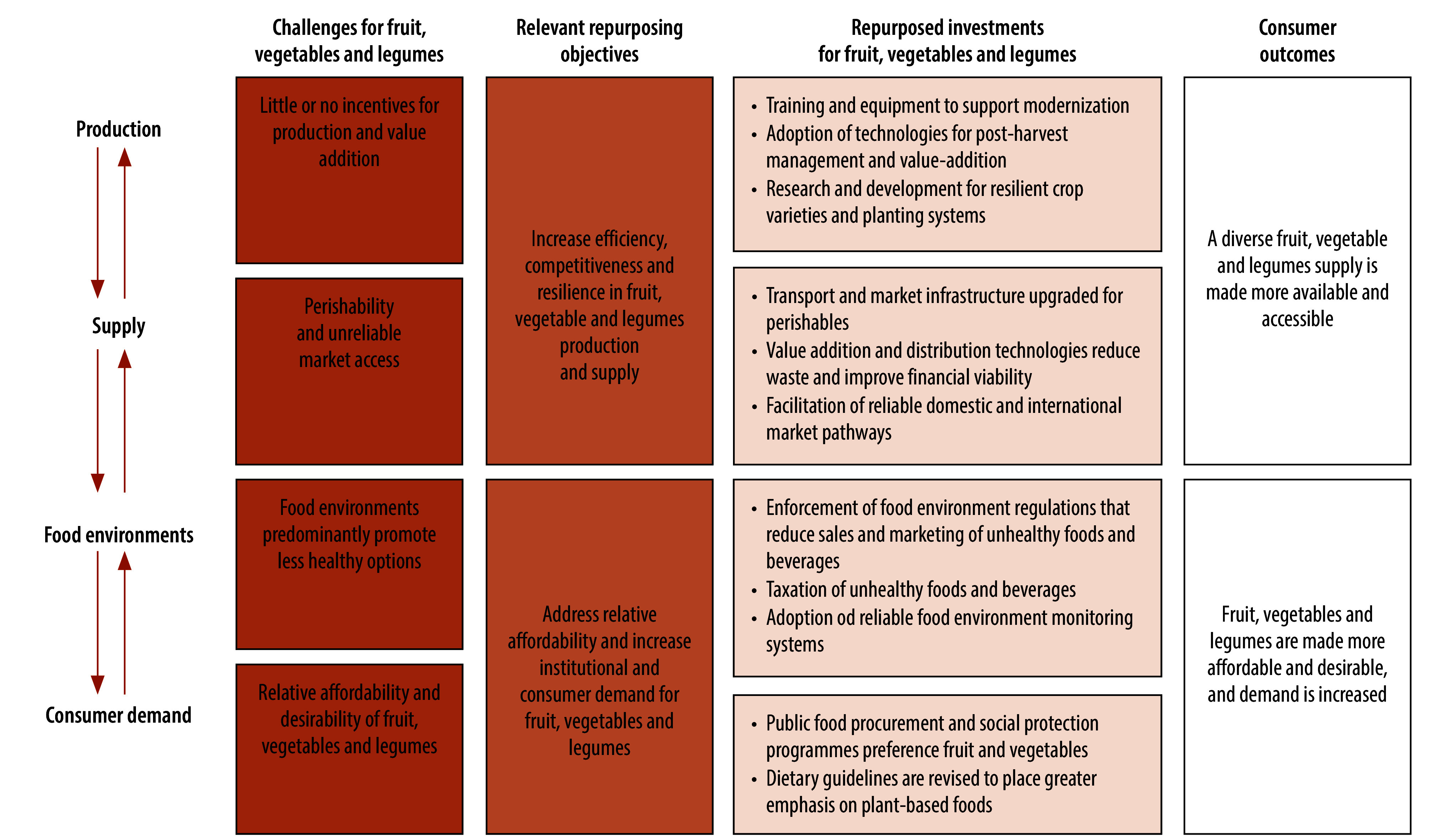
Repurposing agricultural subsidies to promote fruit, vegetable and legumes supply and demand

Enhancing efficiency, competitiveness and resilience in the production and supply of a diverse range of legumes and fresh fruit and vegetables is clearly needed, and so is connecting farmers with reliable market opportunities (Fig. 1).^15^^,^^22^ Many of the initiatives needed to achieve this goal are already being applied by food system sectors with the aim of increasing export production and value addition, and are not designed to target the specific requirements for domestic consumption. An important role for the health policy community is to engage across agriculture, commerce and trade industries on what is needed to reorient these initiatives to address domestic fruit, vegetable and legume production requirements.^2^^,^^22^

Figure reflects priority policy actions to transition food systems towards healthy sustainable diets as they relate to fruit, vegetable and legumes problems and solutions.^36^

The degree of competition across agricultural value chains has a large bearing on determining food prices.^22^ Investments in training and equipment to support modernization could increase the productivity and efficiency of fruit, vegetable and legume producers, providing a valuable opportunity to promote less input-intensive methods.^12^^,^^22^ As fruit and vegetable production is generally more resource intensive than other crop production, investments that minimize resource inputs and consider the protection of natural resources should be considered as priorities.^12^ Investments in research and development of resilient crop varieties and planting systems could improve productivity that preserves soil health without further burdening natural resources,^26^ especially where legumes are used to support nitrogen replenishment and soil regeneration.^25^ Many countries are assessing the potential for vertical farms to produce fruit, vegetables and legumes requiring less water or chemical inputs, and where possible powered by renewable energy.^37^ Ecosystem approaches have been successfully integrated into large-scale legume cropping systems in Australia and the United States of America.^26^ However, accelerating dissemination of ecosystem approaches to legume production in smallholder farm agronomy is needed.^26^

Substantial losses and waste could be averted through strategic investment in the rural infrastructure needed to bring perishable foods to markets efficiently and to store them.^22^^,^^31^ Promising post-harvest technologies exist, including storage, preserving, processing and distribution technologies, that reduce waste or lead to the creation of new value-added products to improve financial viability for producers.^12^ Some niche markets in legume-based starches and pre-soaked legumes have emerged in France that could be replicated to build incentives for farmer participation.^25^

For instance, in India, investments in whole-system approaches to post-harvest management and cold chain development have been found to reduce loss and waste across some crop types by up to 75%.^38^ The Sri Lankan government has been trialling computerized systems for vertical integration between producers, suppliers and distributors, empowering producers to better respond to market demands and emerging opportunities.^20^^,^^39^ Repurposing efforts can emphasize the production of a diverse range or foods and traditional crops that are appropriate to context, and not only those being directed to export trade. The United States have developed adapted crops and soils programmes investing in nutrient-rich traditional and indigenous plants that promote healthy, fertile soils.^40^ Crop-neutral investments like these promote diversification, which is a critical strategy for building resilience, delivering on nutrition outcomes and maximizing ecological performance.^22^^,^^41^

In many settings, governments have positioned production output volumes as the main goal of food systems sectors (known as a productivist paradigm).^31^ However, creating reliable and ongoing linkages between rural farmers and domestic markets (formal and informal) and intraregional and global trade opportunities have proven to be critical for lifting scale and efficiency.^2^^,^^22^ Development of intraregional trade opportunities is currently a major policy agenda in the WHO African Region^42^ and Region of the Americas,^43^ with investments now needed in developing sustainable market pathways that bring perishable produce to market efficiently and in good quality.^22^ Producers having only accessed local markets or sold produce to a middleman may also require procedural support in accessing domestic permits, food risk management and certification.^22^ Health policy-makers can support such linkages by aligning domestic food safety and quality standards to the Codex sanitary and phytosanitary standards to make participation more efficient for producers, particularly smallholders who find it difficult to navigate and fund certification.^22^

### Increasing demand 

The repurposing agenda represents an opportunity to address chronic underinvestment in the design, implementation and enforcement of policies to promote healthy food environments and shape healthy consumption (Fig. 1).^9^ Food-based dietary guidelines have an important role in guiding dietary choices and policy-makers’ priorities; however, without strong food environment policies, these guidelines have limited effect on food patterns.^44^ Additionally, food-based dietary guidelines are not always in alignment with WHO guidance to increase fruit, vegetable and legume consumption, and do not sufficiently convey to consumers the social costs of food choices.^36^ Countries should consider how these guidelines could more strongly build demand for fruit, vegetables and legumes within the context of a more sustainable pattern of consumption,^36^ and then invest in strategies to enhance their uptake. Food-based dietary guidelines that more strongly convey the need to increase fruit, vegetable and legume intake would form a stronger basis for WHO recommendations to regulate unhealthy food and non-alcoholic beverages sales and marketing, and fiscal policies that promote the relative affordability of healthy foods.^9^ The German government has recently updated the national dietary guidelines recommending that people consume at least 75% plant-based diets, while the Danish government has released a national action plan for increasing the affordability of plant-based foods, and for fostering a cultural shift for their demand.^45^ Countries in the Region of the Americas have adopted national dietary guidelines emphasizing unprocessed plant-based foods as the basis of a range of food environment policies. For instance, Brazil’s National School Feeding Programme, underpinned by the country’s 2017 dietary guidelines, has been successful in increasing fruit and vegetable consumption among children.^46^

The purchasing power of governments also represents an important opportunity to build demand for fruit, vegetables and legumes and influence agrifood practices, with public procurement representing 12–20% of total gross domestic product for many countries.^2^ Health policy-makers can scale up efforts to advocate for publicly funded settings and initiatives in which healthy food is the only option,^12^ and social spending (for example vouchers and school breakfast programmes) that emphasizes fruit, vegetable and legume consumption. Strategic sourcing of food by governments can provide fruit, vegetable and legume producers with predictable demand and enhance their living wages.^22^^,^^47^ Brazil has exceeded its targets for over 30% of food in the National School Feeding Programme to be sourced from family farms,^46^ and the approach is now being adapted for use in Malawi and Senegal.^48^

## Building acceptability 

Repurposing agricultural subsidies would shift resources away from one constituency group and towards others, which in many cases will result in trade-offs that may create resistance.^18^^,^^22^ For instance, a shift away from more intensive commodities will likely affect business and livelihood outcomes associated with their production, and maintaining affordable healthy food prices while ensuring that producers are adequately resourced might be challenging.^18^^,^^22^ Private actors benefitting from the current system are likely to be exerting pressure on politicians and policy-makers to maintain the same incentive structures.^4^^,^^49^ Therefore, determining which food systems challenges should be prioritized or ignored is likely to be a sensitive issue.^50^ Those government sectors administering the current subsidy system, including agriculture, rural livelihoods, finance, industry, commerce and trade, are likely to hold greater priority for productivity, value addition, export and food sufficiency than to dietary diversity and population nutrition.^49^

A whole-of-government approach will be critical for operationalizing a wholesale change across food system subsidy systems.^49^ Health policy-makers can help the agricultural sector with navigating the administrative and political economy dimensions of the repurposing agenda by strengthening cooperation with other food system sectors and ensuring their concerns have been considered.^2^^,^^17^ The health sector has experience in engaging across governments and in mobilizing civil society, having advocated for cross-sectoral noncommunicable disease prevention for the past two decades.

The repurposing agenda has emerged from growing concerns for the combined societal impacts of agrifood systems on health, environments and livelihoods.^36^ The agenda can be positioned within the context of a common set of goals for nutrition and global agriculture with proven potential to positively affect the development priorities over which the food system sectors are responsible. Lessons from the adoption of sugar-sweetened beverage taxes around the world include that early and ongoing engagement across government is critical for building support, and that constructive engagement both emphasizes common objectives and addresses concerns.^15^^,^^17^ Such engagement could spur discussion on complementary policy opportunities – for instance, ways that trade policies could better incentivize the reflection of non-market values (such as health or natural resources) in fruit, vegetable and legume production, and integrate measurable targets related to these values.^22^ Global commitments, including the United Nations sustainable development goals, have proved critical for spurring widespread agricultural reforms in the past.^49^

## Conclusions

The current willingness of the agricultural policy community to reallocate food systems subsidies offers an opportunity for health policy-makers to strategically engage in repurposing these subsidies for fruit, vegetables and legumes. Investments are needed to enhance efficiency, competitiveness and resilience in the production and supply of a diverse range of fresh fruit and vegetables. Investments should also be directed towards the implementation and enforcement of food environment policy measures that will increase both consumer and institutional demand for fruit and vegetables. The health sector plays an important role in drawing attention to the need, and in strategically engaging with agricultural policy-makers in setting an agenda that can meaningfully achieve that goal. Positioning the agenda within the context of a common set of goals for nutrition and global agriculture – and emphasizing these throughout engagement with stakeholders – will be key. 
